# Chemical Constituents of Supercritical Extracts from *Alpinia officinarum* and the Feeding Deterrent Activity against *Tribolium castaneum*

**DOI:** 10.3390/molecules22040647

**Published:** 2017-04-18

**Authors:** Mintong Xin, Shanshan Guo, Wenjuan Zhang, Zhufeng Geng, Junyu Liang, Shushan Du, Zhiwei Deng, Yongyan Wang

**Affiliations:** 1 Beijing Key Laboratory of Traditional Chinese Medicine Protection and Utilization, Faculty of Geographical Science, Beijing Normal University, Beijing 100875, China; 201231190019@mail.bnu.edu.cn (M.X.); guoshanshan@mail.bnu.edu.cn (S.G.); zwj0729@mail.bnu.edu.cn (W.Z.); gengzhufeng@bnu.edu.cn (Z.G.); liangjunyu@nwnu.edu.cn (J.L.); narcissus09@126.com (Y.W.); 2 Analytical and Testing Center, Beijing Normal University, Beijing 100875, China; dengzw@bnu.edu.cn

**Keywords:** *Alpinia officinarum*, 1-phenyl-4-(16,17-dimethyl-9,13-octadiene)-5-isopentenyl-7-(4”-methoxyl-3”-hydroxyl-phenyl)-3-heptanone, *Tribolium castaneum*, contact toxicity, feeding deterrent activity

## Abstract

*Alpinia officinarum* has been confirmed to possess bioactivities against some pests. In this work, a sample was obtained from *A. officinarum* rhizomes by supercritical fluid CO_2_ extraction (SFE). According to GC-MS analysis, the main chemical components for SFE-sample included benzylacetone (26.77%), 1,7-diphenyl-5-hydroxy-3-heptanone (17.78%), guaiacylacetone (10.03%) and benzenepropanal (7.42%). The essential oil of *A. officinarum* rhizomes (LD_50_ = 20.71 μg/adult) exhibited more contact toxicity than SFE extract (LD_50_ = 82.72 μg/adult) against *Tribolium castaneum*. From SFE extracts, one new compound, 1-phenyl-4-(16,17-dimethyl-9,13-octadiene)-5-isopentenyl-7-(4”-methoxyl-3”-hydroxyl-phenyl)-3-heptanone (**3**), together with five known compounds identified as 5-hydroxy-1,7-diphenyl-3-heptanone (**1**), 1,7-diphenyl-4-hepten-3-one (**2**), galangin (**4**), galangin-3-methyl ether (**5**) and pinocembrin (**6**), were isolated and their feeding deterrent activities against *T. castaneum* adults were assessed. It was found that compounds **1**–**6** had feeding deterrent activities against *T. castaneum* with feeding deterrent indices of 18.21%, 18.94%, 19.79%, 26.99%, 20.34%, and 35.81%, respectively, at the concentration of 1500 ppm. Hence, the essential oil and SFE extracts/compounds of *A. officinarum* rhizomes represent promising alternatives in the control of *T. castaneum* adults.

## 1. Introduction

It is estimated that direct and indirect losses of grains and grain-based products caused by stored-product insects range from about 10% in temperate regions to almost 50% in humid tropical areas [1]. The red flour beetle, *Tribolium castaneum* (Herbst), a big threat to stored products, has caused serious damage to crop stores throughout the world, including reducing the quantity and quality of the food economy [2,3,4,5]. Several chemical insecticides have been used to protect stored products from insect infestation. Although effective, their repeated use has led to the resurgence and resistance of these insects, resulted in the development of environmental pollution, and produced undesirable effects on non-targeted animals [6,7]. In premodern China, many medicinal herbs and spices were used to control storage pests [8]. Antagonistic storage is a traditional Chinese medicinal material conservation method that has also been used for medicinal materials that have special volatile odors to prevent other Chinese medicinal materials from suffering insect attacks [9]. This method has been in use for a long time, playing an important role in the ecological protection and utilization of traditional Chinese medicine resources [10]. In order to develop this traditional method of prevention and to control storage pests, this study established a screening program and focus on the volatile substances due to their major role in the antagonistic storage process.

*Alpinia officinarum* Hance (Zingiberaceae), commonly known as lesser galangal, is an important plant from the ginger family that originates in southern China and is cultivated in Southeast Asia [11]. Its rhizomes, which have a strong aromatic odour, have been used as Chinese folk medicine (usually called “Gao-liang-jiang”) for decades [12]. Numerous studies reported that *A. officinarum* possesses anti-inflammatory, anticancer, antibacterial, antifungal, antihyperlipidemic, antiemetic, and diuretic properties [11,12,13]. As a traditional Chinese material that is usually used for antagonistic storage to prevent insects [14], *A. officinarum* (especially its essential oil) has been confirmed to possess bioactivities against *Sitophilus zeamais*, *T. castaneum* [15], *Liposcelis bostrychophila* [16], *Lasioderma serricorne* [17], *Coptotermes gestroi*, *Coptotermes curvignathus*, and other pests [18].

Previous research has already investigated the essential oil of *A. officinarum* extracted by hydrodistillation (HD) against *L. serricorne* [17]. Compare with HD, the supercritical fluid CO_2_ extraction (SFE) is an environmentally benign and mild method that may reduce thermal degradation and retain the components without any change. In addition, this method has an economic advantage because it is much faster than other liquids extraction [19,20]. Researchers have investigated the composition of *A. officinarum* by SFE and detected some diarylheptanoids [21]. However, there are few reports available on the bioactivities of the oil extracts and its individual compounds from *A. officinarum* obtained by SFE against *T. castaneum*. The objectives of this study were to (i) compare the contact toxicity of the essential oil and SFE extracts of *A. officinarum* against *T. castaneum* adults; (ii) isolate compounds from SFE section of *A. officinarum*; and (iii) test feeding deterrent activity of the isolated compounds against *T. castaneum*.

## 2. Results and Discussion

### 2.1. Chemical Compounds

The yields of *A. officinarum* samples obtained by HD and SFE were 0.62% (*v*/*w*) [17] and 11.1% (*v*/*w*), respectively. Comparing the two methods, the yield of SFE-sample is much higher than the essential oil because supercritical fluids diffusivities are much faster than in liquids. In addition, due to the lack of surface tension and negligible viscosities compared to liquids, the solvent can penetrate into the matrix to a degree inaccessible to liquids [22]. As shown in Table 1, the results of GC-MS analysis between essential oil and SFE-sample were different. Examination of SFE-sample by GC-MS analysis revealed the presence of 30 components, accounting for 68.90% of the total. The main compounds were identified as benzylacetone (26.77%), 1,7-diphenyl-5-hydroxy-3-heptanone (17.78%), guaiacylacetone (10.03%), and benzenepropanal (7.42%). In the case of essential oil, the GC-MS analysis revealed 31 components representing 69.36%, and the major compounds were identified as 1,8-cineole (51.46%), *α*-terpineol (9.85%), and *δ*-cadinene isomers (5.44%). Most of the main compounds in the SFE sample (1,7-diphenyl-5-hydroxy-3-heptanone, guaiacylacetone, and benzenepropanal) were not identified in the essential oil. This may be because some compounds with low boiling points were removed or because some compounds sensitive to heat changed under a relative high temperature during the long extraction time. In addition, the SFE method was performed using low polarity CO_2_: thus the low polarity constituent (usually with the carbanyl and aldehyde groups) such as aldehydes, ketones, esters, and ethers were more likely to be obtained. Previous research reported the comparison among three *A. officinarum* samples extracted by HD, solvent extraction, and ultrasonic-assisted extraction (UAE) [23]. It was found that the UAE method of obtaining chemical constitutes of *A. officinarum* oil was more efficiency than other two methods, more time saving, and more energy efficient, but the oil quality was poorer due to the use of organic solvents that were hard to remove. Therefore, extraction methods must be selected according to the functional requirements of products.

### 2.2. Structure Elucidation

**Figure 1:**
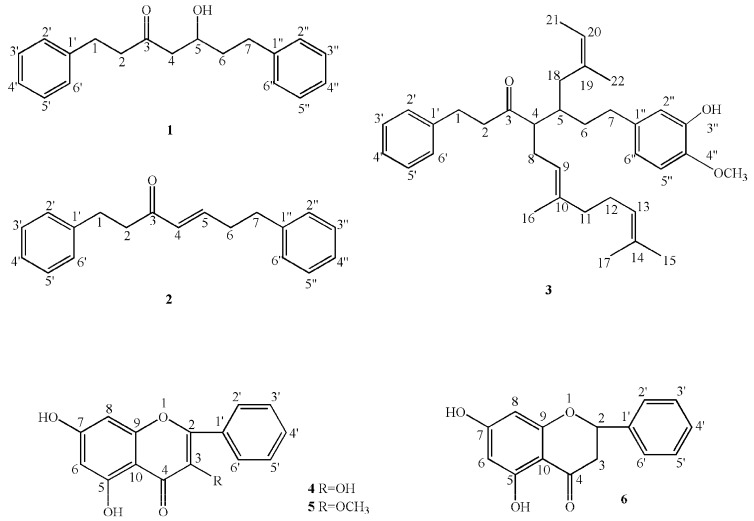
Compounds isolated from *A. officinarum* rhizomes extracted by supercritical fluid CO_2_ extraction (SFE).

From the data in Table 1, it was found that nearly 30% of the compounds were not identified by GC-MS; this may be because those compounds were thermally decomposed by the heating process in testing [21]. Thus, this study tried to isolate the compounds from the SFE extracts of *A. officinarum* rhizomes. Finally, one new (**3**) and five known (**1**–**2**, **4**–**6**) compounds were obtained; their molecular structures are given in Figure 1. The assignment of the ^1^H- and ^13^C-NMR signals of new compounds are listed below in *3.6. Identification of the Compounds*. The new compounds were illustrated by mass spectra and NMR spectra, including ^1^H spectroscopy, ^13^C spectroscopy, ^1^H-^1^H correlation spectroscopy (COSY), heteronuclear single quantum coherence (HSQC), and heteronuclear multiple-bond correlation (HMBC). HRESI-MS and NMR data relating to the new compound (**3**) is available in Figures S1–S6. By matching with the corresponding data (^1^H- and ^13^C-NMR data) in literatures [24,25,26], the five known compounds were determined to be 5-hydroxy-1,7-diphenyl-3-heptanone (**1**), 1,7-diphenyl-4-hepten-3-one (**2**), galangin (**4**), galangin-3-methyl ether (**5**), and pinocembrin (**6**).

**Figure 2:**
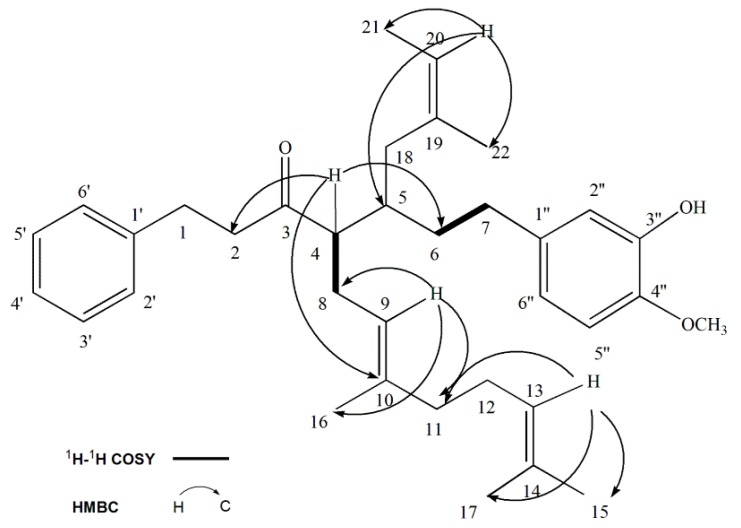
^1^H-^1^H COSY and key HMBC correlation of compound **3**.

Compound **3** isolated as a colorless needle crystals was determined as C_35_H_48_O_3_ by HR-ESI-MS at *m*/*z* 515.3470 [M − H]^−^ (calcd. for C_35_H_4__7_O_3_, 515.3525). The ^1^H-NMR and ^13^C-NMR spectroscopic data of compound **3** were very similar to those of diarylheptanoids skeleton [24,25,26]. In the ^1^H-NMR spectrum, the presence of the phenolic hydroxyl group was supported by active hydrogen. One signal at δ 5.45 (1H, s), and the methoxy group signal was at δ 3.90 (3H, s). The remainder of the spectrum consisted of two sets of signals systems. One set of signals appeared at δ 7.23 (2H, d, *J* = 7.3 Hz, H-2’, 6’), δ 7.16 (3H, t, *J* = 6.5 Hz, H-3’, 4’, 5’) and another at δ 6.82 (1H, d, *J* = 8.0 Hz, H-5”), δ 6.70 (1H, s, H-2”), δ 6.65 (1H, d, *J* = 8.0 Hz, H-6”). Instead of the olefinic proton at δ 5.33 (1H, s, H-20), δ 4.97 (1H, t, *J* = 7.0 Hz, H-9) and δ 5.08 (1H, t, *J* = 7.0 Hz, H-13), the ^1^H-NMR spectrum showed signals for five methyl singlet protons observed at δ 1.72 (3H, s, H-22), δ 1.66 (3H, s, H-17), δ 1.58 (3H, s, H-15), δ 1.53 (3H, s, H-16) and δ 1.27 (3H, s, H-21). The ^13^C spectrum showed the presence of 35 carbons, including one carbonyl carbon at δ 211.3 (C-3), six olefin carbons at δ 120.7 (C-20), δ 123.8 (C-9), δ 124.5 (C-13), δ 131.5 (C-14), δ 135.9 (C-10), δ 136.3 (C-19), five methyl carbons at δ 16.3 (C-16), δ17.8 (C-15), δ25.9 (C-17), δ29.6 (C-21), δ22.9 (C-22), and one methoxyl carbon at δ 56.1 (-OCH_3_). The signal assignment of 2D-NMR spectra was carried out and is shown in Figure 2. The COSY spectrum exhibited the correlations between H-6 (δ 1.80) and H-7 (δ 2.51), and H-4 (δ 2.63) and H-8 (δ 2.24). The HMBC spectrum indicated that δ 6.65 (1H, d, *J* = 8.0 Hz, H-6”) has correlation with 32.9 (C-7), 114.3 (C-5”) and 146.5 (C-4”); δ 6.82 (1H, d, *J* = 8.0 Hz, H-5”) has correlation with 111.1 (C-6”), 134.5 (C-1”), 146.5 (C-4”) and 143.7 (C-3”); δ 6.70 (1H, s, H-2”) has correlation with 134.5 (C-1”), 32.9 (C-7), 146.5 (C-4”) and 143.7 (C-3”); δ 5.08 (1H, t, *J* = 7.0 Hz, H-13) has correlation with 25.9 (C-17),17.8 (C-15) and 40.1 (C-11); δ 4.97 (1H, t, *J* = 7.0 Hz, H-9) has correlation with 40.1 (C-11), 16.3 (C-10) and 28.7 (C-8); δ 5.33 (1H, s, H-20) has correlation with 29.6 (C-21), 22.9 (C-22) and 41.2 (C-5); δ 2.63 (1H, dd, *J* = 4.5, 10.0 Hz, H-4) has correlation with 30.1 (C-8), 211.3 (C-3), 36.5 (C-6), 41.2 (C-5), 28.7 (C-8), 44.9 (C-2) and 135.9 (C-10); δ 7.23 (2H, d, *J* = 7.31 Hz, H-2*’*, 6*’*) has correlation with 29.8 (C-1), 141.5 (C-1*’*) and 128.5 (C-2*’*, 6*’*); δ 2.24 (2H, dd, *J* = 5.9, 11.0 Hz, H-8) has correlation with 28.7 (C-8), 123.8 (C-9), 55.5 (C-4), 211.3 (C-3), 120.7 (C-20), 22.9 (C-22) and 136.3 (C-19). In addition, the quaternary carbons of 4” and 3” (146.5 and 143.7 ppm, respectively) suggested the existence of substituent group. In inductive effect, the -OCH_3_ is a withdrawing electron group stronger than -OH, which leads to the increasing ^13^C chemical shift, so the -OCH_3_ was contacted to 4” while -OH was jointed at 3”. On the basis of these results, the structure of compound **3** was identified as 1-phenyl-4-(16,17-dimethyl-9,13-octadiene)-5-isopentenyl-7-(4”-methoxyl-3”-hydroxyl-phenyl)-3-heptanone.

### 2.3. Contact Toxicity

In Table 2, the essential oil and SFE extracts of *A. officinarum* exhibited contact toxicity against *T. castaneum* adults with LD_50_ values of 20.71 μg/adult and 82.72 μg/adult, respectively. Obviously, the essential oil exhibited much stronger contact toxicity against *T. castaneum* adults than that of SFE-sample, while much less contact toxicity than the positive control, pyrethrins (0.26 μg/adult). Compared with SFE-sample (9.69%), the higher contact toxicity of the essential oil (81.84%) might be attributed to the high content of monoterpenoids. They often play a key role in contact toxicities: for example, 1,8-cineole (LD_50_ = 18.83 μg/adult) and terpinen-4-ol (LD_50_ = 19.67 μg/adult) exhibited strong contact toxicity against *T. castaneum* [27,28]. From this perspective, it would be better to use essential oil than SFE extracts to control *T. castaneum*.

### 2.4. Feeding Deterrent of the Isolated Compounds

The results of feeding deterrent activity of six compounds—5-hydroxy-1,7-diphenyl-3-heptanone (**1**), 1,7-diphenyl-4-hepten-3-one (**2**), 1-phenyl-4-(16,17-dimethyl-9,13-octadiene)-5-isopentenyl-7-(4”-methoxyl-3”-hydroxyl-phenyl)-3-heptanone (**3**), galangin (**4**), galangin-3-methyl ether (**5**), and pinocembrin (**6**)—isolated from SFE-sample of *A. officinarum* rhizome are shown in Table 3. All the isolated compounds showed mild feeding deterrent activity against *T. castaneum* adults. With the increase of concentration, their effects enhanced gradually. At 1500 ppm concentrations, compound **6** (pinocembrin) exhibited the highest feeding deterrent activity among six isolated compounds. In previous research, galangin showed feeding deterrent activity on *Epiphyas postvittana* [30]. It was also found that pinocembrin presents feeding deterrent activity against *Epilachna paenulata* and *Spodoptera frugiperda* [31,32]. This study found feeding deterrent activity from pinocembrin (35.81%, at 1500 ppm concentrations) against *T. castaneum* adults. Compared with other testing concentrations (15–500 ppm), the new compound **3** exhibited relative feeding deterrent activity at highest testing concentrations (1500 ppm). The compounds from SFE extracts also have potential to prevent feeding by this insect. Further research will concentrate on more feeding deterrent activity constituents from *A. officinarum*.

## 3. Materials and Methods

### 3.1. Chemicals

*n*-Hydrocarbons (C_5_–C_36_) were purchased from Sigma-Aldrich (St. Louis, MO, USA). All other chemicals and reagents were of analytical grade.

### 3.2. Plant Material

*A. officinarum* rhizomes were collected from Zhanjiang City, Guangdong, China (20.33° N latitude; 110.17° E longitude). The plant was identified by Dr. Liu, Q.R. (College of Life Science, Beijing Normal University, Beijing, China). A voucher specimen (BNU-CMH-Dushushan-2013-06-11-014) was deposited at the Herbarium of College of Resources Science and Technology in Beijing Normal University.

### 3.3. Extraction and Isolation

*A. officinarum* rhizomes (10 kg) were extracted at 50 °C, 5–6 Mpa, and 30 L/h of CO_2_ flow on HA321-50-16 instrument, for 2.5 h to get SFE-sample. Anhydrous sodium sulphate was used to remove extra water from extraction. The essential oil sample was from previous research [17]. The two samples were stored in airtight containers in a refrigerator at 4 °C for further analysis.

The SFE-sample (30.0 g) was chromatographed on a silica-gel (SiO_2_, 50 mm i.d., 600 mm length, 160 to 200 mesh, Qingdao Marine Chemical Plant, Qingdao, Shandong Province, China) by gradient elution with petroleum ether first, then with petroleum ether-ethyl acetate, and finally with ethyl acetate to obtain 20 fractions. The fractions were further purified on silica gel columns until the pure compounds were obtained. Finally, five pure compounds were obtained from fraction 2 and fraction 6. With the monitoring of thin-layer chromatography (TLC, precoated silica gel G plates, Qingdao Marine Chemical Plant, Qingdao, Shandong, China), fraction 2 was eluted with PE-EtOAc 30/1 on silica gel, then further purified by a Sephadex LH-20 column (Pharmacia, Sweden) and recrystallized to get compound **1** (2267.2 mg, Figure 1) and **2** (23.6 mg, Figure 1). Fraction 6 was re-chromatographed on silica gel and eluted with PE-EtOAc, and then on a Sephadex LH-20 column (Pharmacia, Sweden) with methanol to yield compound **3** (17.6 mg, Figure 1), **4** (450.1 mg, Figure 1), **5** (19.0 mg, Figure 1) and **6** (388.0 mg, Figure 1). The molecular structures of the isolated compounds were elucidated based on the analysis of ^1^H- and ^13^C-NMR spectra, which were recorded on an Avance III NMR spectrometer (Bruker-Biospin, Billerica, MA, USA).

### 3.4. Insects

Examples of the red flour beetle *T. castaneum* used in the following screening for the test were obtained from laboratory cultures for the last three years in a dark incubator at 28–30 °C, with 70–80% relative humidity. The insects were reared in glass containers (0.5 L) containing wheat flour at 12–13% moisture content mixed with yeast (wheatfeed/yeast, 10:1, *w*/*w*). Adults used in the experiments were about two weeks old.

### 3.5. GC-MS and GC-FID Analyses

The two samples of *A. officinarum* were analyzed using a Thermo Finnigan Trace DSQ GC/MS instrument (Thermo Finnigan, Lutz, FL, USA) equipped with a flame ionization detector (FID) and a HP-5MS (30 m × 0.25 mm × 0.25 μm) capillary column. The mass spectrometer was performed in the electron-impact mode, with ionization energy of 70 eV in m/e at a range of 10–550 amu. The same column and analysis were used in both GC-FID and GC-MS analysis. The temperature was programmed isothermal at 50 °C for 2 min, then rising from 50 to 150 °C at the speed of 2 °C/min, then held isothermal for 2 min at 150 °C, rising from 150 to 250 °C at the speed of 10 °C/min, and finally held isothermal at 250 °C for 5 min. The injector temperature was 250 °C, and the flow rate of helium (the carrier gas) was 1.0 mL/min. The samples were diluted (1% solution, *v*/*v*, diluted in *n*-hexane) and then manually injected in the split mode. Constituents of the two samples were identified by comparing their retention indices (RIs), which were determined to the retention times of a series of *n*-alkanes (C_5_–C_36_) or with those reported in the literature [33]. Quantification was determined by percentage peak area calculations using GC-FID, while, the major constituents were identified by being coinjected with standards and confirmed by using the National Institute of Standards and Technology (NIST) version 05 GC-MS libraries (Standard Reference Data, Gaithersburg, MD, USA) and Wiley 275 mass-spectral libraries (Wiley, New York, NY, USA) or in the literature [34].

### 3.6. Identification of the Compounds

Chemical structures were assigned by analysis of the MS, ^1^H, ^13^C and 2D-NMR spectra and comparison with literature values. Accordingly, compounds **1**–**6** were identified as 5-hydroxy-1,7-diphenyl-3-heptanone [24,25], 1,7-diphenylhept-4-en-3-heptanone [26], 1-phenyl-4-(16,17-dimethyl-9,13-octadiene)-5-isopentenyl-7-(4”-hydroxyl-3”-methoxyl-phenyl)-3-heptanone, galangin [26], galangin-3-methyl ether [26], and pinocembrin [26], respectively (Figure 1).

*5-Hydroxy-1,7-diphenyl-3-heptanone* (**1**). C_19_H_22_O_2_, colorless needles. ESI-MS *m*/*z*: 283.1 [M + H]^+^. ^1^H-NMR (500 MHz, CDCl_3_) δppm: 7.29 (4H, m, H-2’, 6’, 2”, 6”), 7.20 (6H, m, H-3’, 4’, 5’, 3”, 4”, 5”), 4.06 (1H, dq, *J* = 12.3, 4.0 Hz, H-5), 3.87 (1H, br s, OH), 2.91 (2H, t, *J* = 7.6 Hz, H-1), 2.81 (1H, m, H-2), 2.76 (2H, dd, *J* = 13.6, 5.9 Hz, H-4), 2.68 (1H, ddd, *J* = 13.8, 9.3, 7.1 Hz, H-2), 2.55 (2H, m, H-7), 1.81 (1H, ddd, *J* = 14.2, 8.8, 4.5 Hz, H-6), 1.68 (1H, m, H-6); ^13^C-NMR (125 MHz, CDCl_3_) δ ppm: 211.2 (C-3), 141.9 (C-1’), 140.8 (C-1”), 128. 7 (C-3’, 5’), 128.6 (C-3”, 5”), 128.5 (C-2”, 6”), 128.4 (C-2’, 6’), 126.3 (C-4’), 126.0 (C-4”), 67.0 (C-5), 49.4 (C-4), 45.1 (C-2), 38.1 (C-6), 31.8 (C-1), 29.6 (C-7).

*1,7-Diphenyl-4-hepten-3-one* (**2**). C_19_H_20_O, colorless needles. ESI-MS *m*/*z*: 265.1 [M + H]^+^. ^1^H-NMR (500MHz, CDCl_3_) δ ppm: 7.29 (4H, m, H-3’, 5’, 3”, 5”), 7.19 (6H, m, H-2’, 4’, 6’, 2”, 4”, 6”), 6.84 (1H, dt, *J* = 6.8, 15.9 Hz, H-5), 6.11 (1H, d, *J* = 15.9 Hz, H-4), 2.93 (2H, t, *J* = 7.0 Hz, H-2), 2.84 (2H, dd, *J* = 4.4, 11.2 Hz, H-1), 2.77 (2H, t, *J* = 7.7 Hz, H-7), 2.53 (2H, q, *J* = 7.1 Hz, H-6); ^13^C-NMR (125 MHz, CDCl_3_) δ ppm: 199.6 (C-3), 146.5 (C-5), 141.4 (C-1’), 140.8 (C-1”), 130.9 (C-4), 128.7 (C-3’, 5’), 128.5 (C-2’, 6’), 128.6 (C-3”, 5”), 128.5 (C-2”, 6”), 126.4 (C-4’), 126.2 (C-4”), 41.9 (C-2), 34.3 (C-6), 34.6 (C-7), 30.2 (C-1).

*1-Phenyl-4-(16,17-dimethyl-9,13-octadiene)-5-isopentenyl-7-(4”-methoxyl-3”-hydroxyl-phenyl)-3-heptanone* (**3**). C_35_H_48_O_3_, colorless needles, HRESI-MS m/z: 515.3470 [M − H]^+^. ^1^H-NMR (500 MHz, CDCl_3_) δ ppm: 7.23 (2H, d, *J* = 7.3 Hz, H-2’, 6’), 7.16 (3H, t, *J* = 6.5 Hz, H-3’, 4’, 5’), 6.82 (1H, d, J = 8.0 Hz, H-5”), 6.70 (1H, s, H-2”), 6.65 (1H, d, J = 8.0 Hz, H-6”), 5.45 (1H, s, 4”-OH), 5.33 (1H, s, H-20), 5.08 (1H, t, *J* = 7.0 Hz, H-13), 4.97 (1H, t, *J* = 7.0 Hz, H-9), 3.90 (3H, s, 3”-OCH_3_), 2.83 (2H, m, H-1), 2.78 (2H, m, H-2), 2.63 (1H, dd, *J* = 4.5, 10.0 Hz, H-4), 2.51 (2H, ddd, *J* = 5.5, 10.5, 16.5 Hz, H-7), 2.24 (2H, dd, *J* = 5.9, 11.0 Hz, H-8), 2.06 (5H, m, H-18), 1.96 (1H, m, H-11), 1.80 (1H, m, H-6), 1.72 (3H, s, H-22), 1.66 (3H, s, H-17), 1.58 (3H, s, H-15), 1.53 (3H, s, H-16), 1.27 (3H, s, H-21); ^13^C-NMR (125 MHz, CDCl_3_) δ ppm: 211.3 (C-3), 146.5 (C-4”), 143.7 (C-3”), 141.5 (C-1’), 136.3 (C-19), 135.9 (C-10), 134.5 (C-1”), 131.5 (C-14), 128.6 (C-3’, 5’), 128.5 (C-2’, 6’), 126.1 (C-4’), 124.5 (C-13), 123.8 (C-9), 121.0 (C-2”), 120.7 (C-20), 114.3 (C-5”), 111.1 (C-6”), 56.1 (3”-OCH_3_), 55.5 (C-4), 44.9 (C-2), 41.2 (C-5), 40.1 (C-11), 36.6 (C-12)，36.5 (C-6), 32.9 (C-7), 30.1 (C-8), 29.8 (C-1), 29.6 (C-21), 28.7 (C-8), 26.8 (C-12), 25.9 (C-17), 22.9 (C-22), 17.8 (C-15), 16.3 (C-16).

*Galangin* (**4**). C_15_H_10_O_3_, yellow needles. ESI-MS *m*/*z*: 269.0 [M − H]^−^. ^1^H-NMR (500 MHz, DMSO-*d*_6_) δ ppm: 12.36 (1H, s, 5-OH), 10.86 (1H, s, 7-OH), 9.67 (1H, s, 3-OH), 8.15 (2H, d, *J* = 7.8 Hz, H-2’, 6’), 7.55 (2H, t, *J* = 7.5 Hz, H-3’, 5’), 7.50 (1H, t, *J* = 7.2 Hz, H-4’), 6.47 (1H, s, H-8), 6.21 (1H, s, H-6); ^13^C-NMR (125 MHz, DMSO-*d*_6_) δ ppm: 177.7 (C-4), 165.9 (C-7), 162.7 (C-9), 158.5 (C-10), 147.0 (C-1’), 138.5 (C-2), 132.7 (C-3’,5’), 128.8 (C-3), 130.9 (C-4’), 129.5 (C-2’,6’), 104.7 (C-10), 99.4 (C-6), 94.5 (C-8).

*Galangin-3-methyl ether* (**5**). C_16_H_12_O_3_, yellow powder. ESI-MS *m*/*z*: 283.0 [M − H]^−^. ^1^H-NMR (500 MHz, DMSO-*d*_6_) δ ppm: 12.57 (1H, s, 5-OH), 10.92 (1H, s, 7-OH), 8.01 (2H, dd, *J* = 3.0, 6.6 Hz, H-2’, 6’), 7.58 (3H, m, H-3’, 4’, 5’), 6.47 (1H, d, *J* = 2.0 Hz, H-8), 6.23 (1H, d, *J* = 1.9 Hz, H-6), 3.81 (3H, s, 3-OCH_3_); ^13^C-NMR (125 MHz, DMSO-*d*_6_) δ ppm: 178.1 (C-4), 164.4 (C-7), 161.3 (C-5), 156.6 (C-9), 155.2 (C-2), 138.8 (C-3), 131.1 (C-4’), 130.1 (C-1’), 128.8 (C-3’, 5’), 128.2 (C-2’, 6’), 104.5 (C-10), 98.7 (C-6), 93.9 (C-8), 60.0 (3-OCH_3_).

*Pinocembrin* (**6**). C_15_H_12_O_2_, white needles. ESI-MS *m*/*z*: 255.0 [M − H]^−^. ^1^H-NMR (500 MHz, DMSO-*d*_6_) δ ppm: 12.13 (1H, s, 5-OH), 10.82 (1H, s, 7-OH), 7.52 (2H, d, *J* = 7.5 Hz, H-2’, 6’), 7.41 (3H, dt, *J* = 7.2, 23.3 Hz, H-3’, 4’, 5’), 5.91 (2H, m, H-6, 8), 5.59 (1H, dd, *J* = 2.9, 12.7 Hz, H-2), 3.26 (1H, dd, *J* = 12.7, 17.1 Hz, H-3α), 2.78 (1H, dd, *J* = 2.9, 17.1 Hz, H-3β); ^13^C-NMR (125 MHz, DMSO-*d*_6_) δ ppm: 196.0 (C-4), 166.7 (C-7), 163.5 (C-9), 162.7 (C-5), 138.7 (C-1’), 128.5 (C-3’, 4’, 5’), 126.6 (C-2’, 6’), 101.8 (C-10), 95.9 (C-6), 95.0 (C-8), 78.4 (C-2), 42.1 (C-3).

### 3.7. Contact Toxicity

The contact toxicity of two samples against *T. castaneum* adults was measured as described by Liu and Ho [35]. Previous studies were run to find range and determine the appropriate testing concentrations. The serial dilutions of the oil extracts were prepared in *n*-hexane. Aliquots of 0.5 μL of the dilutions were applied topically to the dorsal thorax of the insects. Controls were determined using *n*-hexane. Both treated and control insects were then transferred to glass vials (10 insects per vial, five replicates per dose) with feed and kept in incubators. Mortality percentages were recorded after 24 h of the treatment and the LD_50_ values were calculated using Probit analysis [36].

### 3.8. Feeding Deterrent Bioassay

A flour-disk bioassay was used to evaluate the feeding deterrent activities of six isolated compounds from rhizomes of *A. officinarum* following the method of Xieal et al. [37] with some modification [35,38,39,40]. The test solutions (1 mg/mL) were prepared with pure compounds dissolved in ethanol. Serials of even flour-water suspensions (2 mL) were prepared with 0.4 g wheat flour, different volumes of the testing solutions, and distilled water. Aliquots (200 μL) of this ultrasonically stirred suspension were placed on the bottom of a polystyrene Petri dish to form disks. About 1 cm was cut from the bottom of a disposable tip with a razor blade to make an opening enlarged to about 2 mm in diameter to make appropriate flour disks. The same amounts of ethanol and distilled water were applied to form the control flour disks. The flour disks were air-dried in the fume-hood overnight. Then, all flour-disks were transferred to an incubator to equilibrate at 28–30 °C and 70–80% R.H. for 48 h. The moisture content of each disk was controlled to be 13.5 ± 0.1% using the Kett’s Grain moisture tester (Model PB-1D2, Tokyo, Japan). The flour disks were placed in glass vials (diameter 2.5 cm, height 5.5 cm) for weighing. Twenty groups were weighed, and then unsexed insects were added to each vial prior for further weighing. All the testing insects were starved for 24 h before use. After that, all the experimental set-up was placed in the incubator for three days. Finally, all the tested insects were picked out and the uneaten parts of the flour disks were weighed. Compared to the control group, the insect consumption for the different test samples was calculated. The percentage feeding deterrent index was calculated from the following equation:

Feeding deterrent index = [(*C* − *T*)*/C*] × 100%

where *C* is the weight of diet consumed in control and *T* is the weight of diet consumed in the treatment. Five replicates of each concentration of each compound and control were done for the test.

## 4. Conclusions

This work compared the chemical composition of two samples of *A. officinarum* rhizomes obtained by HD and SFE method and their contact toxicity on *T. castaneum* adults. The different toxicity attributed to their various chemical constitutes. Extraction methods must be selected according to the functional requirements of products. One new compound (**3**) together with five known compounds (**1**–**2**, **4**–**6**) was isolated from the SFE sample. All the isolated compounds exhibited mild feeding deterrent activities against *T. castaneum* adults (15–1500 ppm). The new compound showed feeding deterrent activities (19.79%) at 1500 ppm. Compound **6** (pinocembrin) had strong feeding deterrent activities (35.81%) against *T. castaneum* at the concentration of 1500 ppm. These results showed that the essential oil and SFE extracts/compounds of *A. officinarum* rhizomes are an alternative in the control of stored-product insects. They also provided significant information for the development and comprehensive utilization of *A. officinarum*.

## Figures and Tables

**Table 1:** Chemical components of two samples from *A. officinarum* rhizomes.

Compounds	Molecular Formula	RI ^a^	Relative Content		Identification Methods ^c^
			Essential Oil ^b^	SFE Extracts	
*α*-Pinene	C_10_H_16_	940	3.26	-	RI, MS
Camphene	C_10_H_16_	956	4.57	-	RI, MS, Co
Sabinene	C_10_H_16_	976	3.65	0.14	RI, MS, Co
*β*-Pinene	C_10_H_16_	978	-	0.09	RI, MS, Co
*α*-Phellandrene	C_10_H_16_	1005	0.49	0.13	RI, MS
*β*-Phellandrene	C_10_H_16_	1026	3.42	-	RI, MS
1,8-Cineole	C_10_H_18_O	1031	51.64	0.80	RI, MS, Co
*γ*-Terpinene	C_10_H_16_	1057	0.67	0.19	RI, MS, Co
Isoterpinolene	C_10_H_16_	1085	0.23	0.13	RI, MS
Linalool	C_10_H_18_O	1099	0.28	0.06	RI, MS
Camphor	C_10_H_18_O	1145	1.84	0.05	RI, MS, Co
Camphene hydrate	C_10_H_18_O	1152	0.16	-	RI, MS
Borneol	C_10_H_18_O	1159	0.38	-	RI, MS
Benzenepropanal	C_9_H_10_O	1167	-	7.42	RI, MS
Terpinen-4-ol	C_10_H_18_O	1177	1.4	-	RI, MS, Co
*α*-Terpineol	C_10_H_18_O	1191	9.85	0.68	RI, MS, Co
Benzylacetone	C_10_H_12_O	1211	0.53	26.77	RI, MS
Fenchyl acetate	C_12_H_20_O_2_	1218	0.55	-	RI, MS
Nonanoic acid	C_9_H_18_O_2_	1283	-	0.35	RI, MS
*α*-Cubebene	C_15_H_24_	1352	0.50	-	RI, MS
*α*-Terpinyl acetate	C_12_H_20_O_2_	1360	-	0.24	RI, MS
*α*-Copaene	C_15_H_24_	1372	0.45	-	RI, MS
Isoledene	C_15_H_24_	1375	0.38	-	RI, MS
*β*-Elemene	C_15_H_24_	1388	0.37	-	RI, MS
*α*-Bergamotene	C_15_H_24_	1410	-	0.56	RI, MS
*α*-Santalol	C_15_H_24_O	1417	-	0.34	RI, MS
*β*-Caryophyllene	C_15_H_24_	1420	0.44	0.07	RI, MS
Undecanoic acid	C_11_H_22_O_2_	1441	-	0.27	RI, MS
*α*-Humulene	C_15_H_24_	1455	0.13	-	RI, MS, Co
Alloaromadendrene	C_15_H_24_	1463	-	0.12	RI, MS
*β*-Patchoulene	C_15_H_24_	1465	0.41	-	RI, MS
Germacrene D	C_15_H_24_	1480	1.13	-	RI, MS
*β*-Selinene	C_15_H_24_	1485	0.14	0.05	RI, MS
Valencene	C_15_H_24_	1489	-	0.09	RI, MS
*α*-Selinene	C_15_H_24_	1492	1.62	0.05	RI, MS
*α*-Muurolene	C_15_H_24_	1497	0.34	-	RI, MS
Zingiberene	C_15_H_24_	1498	1.05	-	RI, MS, Co
Calamenene	C_15_H_2__2_	1504	0.42	-	RI, MS
*δ*-Cadinene isomers	C_15_H_24_	1523	5.44	0.42	RI, MS
Guaiacylacetone	C_10_H_12_O_3_	1528	-	10.13	RI, MS
Viridiflorol	C_15_H_26_O	1588	-	1.42	RI, MS
*τ*-Muurolol	C_15_H_26_O	1643	-	0.04	RI, MS
*β*-Eudesmol	C_15_H_26_O	1648	-	0.03	RI, MS
*α*-Cadinol	C_15_H_26_O	1654	0.65	-	RI, MS
*Z*-*α*-trans-Bergamotol	C_15_H_24_O	1685	-	0.18	RI, MS
Aristolone	C_15_H_2__2_O	1765	-	0.05	RI, MS
1,7-Diphenyl-5-hydroxy-3-heptanone	C_19_H_20_O	1785	-	17.68	RI, MS
3-Phenylbutanol	C_10_H_14_O	1789	-	0.35	RI, MS
Monoterpenoids			81.84	9.69	
Sesquiterpenoids			13.47	3.42	
Total			96.39	68.90	

^a^ RI, retention index of the chromatography determined on a HP-5MS column using the homologous series of *n*-alkanes as reference. ^b^ The retested data were about the same with our previous results [17]. ^c^ Identification method: RI, comparison of retention indices with published data; MS, comparison of mass spectra with those listed in the NIST 05 and Wiley 275 libraries and with published data; Co, co-injection with standard compound.

**Table 2:** Contact toxicity of two samples from *A. officinarum* rhizomes.

Samples	LD_50_ (μg/Adult)	95% Fiducial Limits	Slope ± SE	Chisquare (χ^2^)
Essential Oil	20.71	2.96–35.85	1.39 ± 0.41	14.22
SEF-sample	82.72	62.28–100.29	1.49 ± 0.28	10.13
Pyrethrins *	0.26	0.22–0.30	3.34 ± 0.32	13.11

* Data from You et al. [29].

**Table 3:** Feeding deterrent activity of six compounds isolated from SFE-sample of *A. officinarum* rhizomes against *T. castaneum* adults.

Compounds	Feeding Deterrent Indices (%) (Mean ± SD)				
	Concentration * (ppm)				
	15	50	150	500	1500
**1**	14.56 ± 1.12	15.16 ± 1.05	16.24 ± 0.68	16.50 ± 3.15	18.21 ± 2.71
**2**	1.13 ± 0.98	6.29 ± 1.25	9.33 ± 0.88	18.09 ± 1.59	18.94 ± 1.38
**3**	12.78 ± 1.30	13.91 ± 1.81	19.12 ± 2.80	19.68 ± 2.75	19.79 ± 2.62
**4**	15.98 ± 2.20	18.10 ± 1.39	19.72 ± 0.75	23.79 ± 2.23	26.99 ± 1.27
**5**	12.84 ± 2.79	13.67 ± 0.82	14.48 ± 1.07	15.68 ± 1.51	20.34 ± 0.78
**6**	10.38 ± 1.75	12.60 ± 1.07	16.09 ± 2.18	19.94 ± 1.32	35.81 ± 2.24

* Concentration and feeding deterrent index of the positive control is 0.

## Supplementary Materials

Supplementary materials are available online. HRESI-MS and NMR data relating to the new compound (**3**) is available in Figure S1–S6.

## References

[1] Wijayaratne L.K.W., Arthur F.H., Whyard S. (2016). Methoprene and control of stored-product insects. J. Stored. Prod. Res..

[2] Buckman K.A., Campbell J.F. (2013). How varying pest and trap densities affect *Tribolium castaneum* capture in pheromone traps. Entomol. Exp. Appl..

[3] Liu Z.L., Cao J., Zhang H.M., Lin L.L., Du S.S., Zhou L.G., Deng Z.W. (2011). Feeding deterrents from *Aconitum episcopale* roots against the red flour beetle, *Tribolium castaneum*. J. Agric. Food Chem..

[4] You C.X., Wang Y., Zhang W.J., Yang K., Wu Y., Geng Z.F., Chen H.P., Jiang H.Y., Du S.S., Deng Z.W. (2014). Chemical constituents and biological activities of the Purple *Perilla* essential oil against *Lasioderma serricorne*. Ind. Crop Prod..

[5] Zettler J.L., Arthur F.H. (2000). Chemical control of stored product insects with fumigants and residual treatments. Crop Prot..

[6] Copping L.G., Menn J.J. (2000). Biopesticides: A review of their action, applications and efficacy. Pest Manag. Sci..

[7] Isman M.B. (2006). Botanical insecticides, deterrents, and repellents in modern agriculture and an increasingly regulated world. Annu. Rev. Entomol..

[8] Ding W., Liu H., Li L.S. (2000). The main stratagems and technology for stored product pest control in ancient China. J. Southwest Agric. Univ..

[9] Yang K., You C.X., Wang C.F., Guo S.S., Li Y.P., Wu Y., Geng Z.F., Deng Z.W., Du S.S. (2014). Composition and repellency of the essential oils of *Evodia calcicola* Chun ex Huang and *Evodia trichotoma* (Lour.) Pierre against three stored product insects. J. Oleo Sci..

[10] Wang C.F., Yang K., You C.X., Zhang W.J., Guo S.S., Geng Z.F., Du S.S., Wang Y.Y. (2015). Chemical composition and insecticidal activity of essential oils from *Zanthoxylum dissitum* leaves and roots against three species of storage pests. Molecules.

[11] Zou Q.Y., Wu H.F., Tang Y.L., Chen D.Z. (2016). A new labdane diterpene from the rhizomes of *Alpinia officinarum*. Nat. Prod. Res..

[12] Lin L.Y., Peng C.C., Yeh X.Y., Huang B.Y., Wang H.E., Chen K.C., Peng R.Y. (2015). Antihyperlipidemic bioactivity of *Alpinia officinarum* (Hance) Farw Zingiberaceae can be attributed to the coexistance of curcumin, polyphenolics, dietary fibers and phytosterols. Food Funct..

[13] Li H.F., Li Y.H., Wang Y., Wei N., Tan Y.F., Zhang J.Q. (2014). Advances in studies on chemical constituents in *Alpiniae officinarum* rhizoma and their pharmacological activities. Chin. J. Exp. Tradit. Med. Form..

[14] Liu C.N. (1987). Antagonistic storage method of traditional Chinese medicinal material. J. Chin. Med. Mater..

[15] Liu Z.L., Goh S.H., Ho S.H. (2007). Screening of Chinese medicinal herbs for bioactivity against *Sitophilus zeamais* Motschulsky and *Tribolium castaneum* (Herbst). J. Stored Prod. Res..

[16] Zhao N.N., Zhou L.G., Liu Z.L., Du S.S., Deng Z.W. (2012). Evaluation of the toxicity of the essential oils of some common Chinese spices against *Liposcelis bostrychophila*. Food Control.

[17] Wu Y., Wang Y., Li Z.H., Wang C.F., Wei J.Y., Li X.L., Wang P.J., Zhou Z.F., Du S.S., Huang D.Y. (2014). Composition of the essential oil from *Alpinia galanga* rhizomes and its bioactivity on *Lasioderma serricorne*. Bull. Insectol..

[18] Abdullah F., Subramanian P., Ibrahim H., Malek S.N.A., Lee G.S., Hong S.L. (2015). Chemical composition, antifeedant, repellent, and toxicity activities of the rhizomes of galangal, *Alpinia galanga* against Asian subterranean termites, *Coptotermes gestroi* and *Coptotermes curvignathus* (Isoptera: Rhinotermitidae). J. Insect Sci..

[19] Conde-Hernández L.A., Espinosa-Victoria J.R., Trejo A., Guerrero-Beltrán J.A. (2017). CO_2_-supercritical extraction, hydrodistillation and steam distillation of essential oil of rosemary (*Rosmarinus officinalis*). J. Food Eng..

[20] Garcia-Perez J.S., Robledo-Padilla F., Cuellar-Bermudez S.P., Parra-Saldivar R., Zavala-Yoe R., Ramirez-Mendoza R.A., Iqbal H.M.N. (2017). Thermodynamics and statistical correlation between supercritical-CO_2_, fluid extraction and bioactivity profile of locally available mexican plants extracts. J. Supercrit. Fluid.

[21] Luo J., Rui W., Jiang M., Tian Q., Ji X., Feng Y. (2010). Separation and identification of diarylheptanoids in supercritical fluid extract of *Alpinia officinarum* by UPLC-MS-MS. J. Chromatogr. Sci..

[22] Sunarso J., Ismadji S. (2009). Decontamination of hazardous substances from solid matrices and liquids using supercritical fluids extraction: A review. J. Hazard. Mater..

[23] Pang Q.H., He J.H., Zeng R.J., Zhao S.J. (2008). Comparison of volatile oil in *Alpinia officinarum* Hance extracted by different methods. Pharm. Biotechnol..

[24] Hema P.S., Nair M.S. (2009). Flavonoids and other constituents from the rhizomes of *Alpinia calcarata*. Biochem. Syst. Ecol..

[25] Pulkkinen J.T., Honkakoski P., Peräkylä M., Berczi I., Laatikainen R. (2008). Synthesis and evaluation of estrogen agonism of diaryl 4,5-dihydroisoxazoles, 3-hydroxyketones, 3-methoxyketones, and 1,3-diketones: A compound set forming a 4d molecular library. J. Med. Chem..

[26] An N., Yang S.L., Zhou Z.M., Xu L.Z. (2006). Flavonoids of *Alpinia officinarum*. Chin. Tradit. Herb. Drugs.

[27] Zhang W.J., Yang K., You C.X., Wang Y., Wang C.F., Wu Y., Geng Z.F., Su Y., Du S.S., Deng Z.W. (2015). Bioactivity of essential oil from *Artemisia stolonifera* (Maxim.) Komar. and its main compounds against two stored-product insects. J. Oleo Sci..

[28] Zhang W.J., Yang K., You C.X., Wang C.F., Geng Z.F., Su Y., Wang Y., Du S.S., Deng Z.W. (2015). Contact toxicity and repellency of the essential oil from *Mentha haplocalyx* Briq.against *Lasioderma serricorne*. Chem. Biodivers..

[29] You C.X., Yang K., Wu Y., Zhang W.J., Wang Y., Geng Z.F., Chen H.P., Jiang H.Y., Du S.S., Deng Z.W. (2014). Chemical composition and insecticidal activities of the essential oil of *Perilla frutescens* (L.) Britt. aerial parts against two stored product insects. Eur. Food Res. Technol..

[30] Russell G.B., Bowers W.S., Keesing V., Niemeyer H.M., Sevenet T., Vasanthaverni S., Wratten S.D. (2000). Patterns of bioactivity and herbivory on Nothofagus species from Chile and New Zealand. J. Chem. Ecol..

[31] Napal G.N.D., Defagó M.T., Valladares G.R., Sara M.P. (2010). Response of *Epilachna paenulata* to two flavonoids, pinocembrin and quercetin, in a comparative study. J. Chem. Ecol..

[32] Georgina N., Napal D., Carpinella M.C., Palacios S.M. (2009). Antifeedant activity of ethanolic extract from *Flourensia oolepis* and isolation of pinocembrin as its active principle compound. Bioresour. Technol..

[33] Huang A., Sefton M.A., Taylor D.K. (2015). Comparison of the formation of peppery and woody sesquiterpenes derived from α-guaiene and α-bulnesene under aerial oxidative conditions. J. Agric. Food Chem..

[34] Adam R.P. (2001). Identification of essential oil components by gas chromatography/quadrupole mass spectroscopy. J. Am. Soc. Mass Spectrom..

[35] Liu Z.L., Ho S.H. (1999). Bioactivity of the essential oil extracted from *Evodia rutaecarpa* Hook f. et Thomas against the grain storage insects, *Sitophilus zeamais* Motsch. and *Tribolium castaneum* (Herbst). J. Stored Prod. Res..

[36] Sakuma M. (1998). Probit analysis of preference data. Appl. Entomol. Zool..

[37] Xieal Y.S., Bodnarykal R.P., Fieldsal P.G. (1996). A rapid and simple flour-disk bioassay for testing substance active against stored-product insects. Can. Entomol..

[38] Du S.S., Wang C.F., Li J., Zhang H.M., Liu Q.Z., Liu Z.L., Deng Z.W. (2011). Antifeedant diterpenoids against *Tribolium castaneum* from the stems and twigs of *Ceriops tagal* (Rhizophoraceae). Molecules.

[39] Liu Z.L., Chu S.S., Jiang G.H. (2009). Feeding Deterrents from *Zanthoxylum schinifolium* against two stored-product insects. J. Agric. Food Chem..

[40] Wang C.F., You C.X., Yang K., Guo S.S., Geng Z.F., Fan L., Du S.S., Deng Z.W., Wang Y. (2015). Antifeedant activities of methanol extracts of four *Zanthoxylum* species and benzophenanthridines from stem bark of *Zanthoxylum schinifolium* against *Tribolium castaneum*. Ind. Crop. Prod..

