# Supplementary material for: Chemical Constituents of Supercritical Extracts from Alpinia officinarum and the Feeding Deterrent Activity against Tribolium castaneum
Source: Molecules. 2017 Apr 18;22(4):647. doi: 10.3390/molecules22040647 (PMC6154697; doi:10.3390/molecules22040647)
Supplement: Supplementary file 1 [file molecules-22-00647-s001.pdf]

Supplementary File. The MS, ^{1}H, ^{13}C and 2D NMR spectra of the new compound **3**.

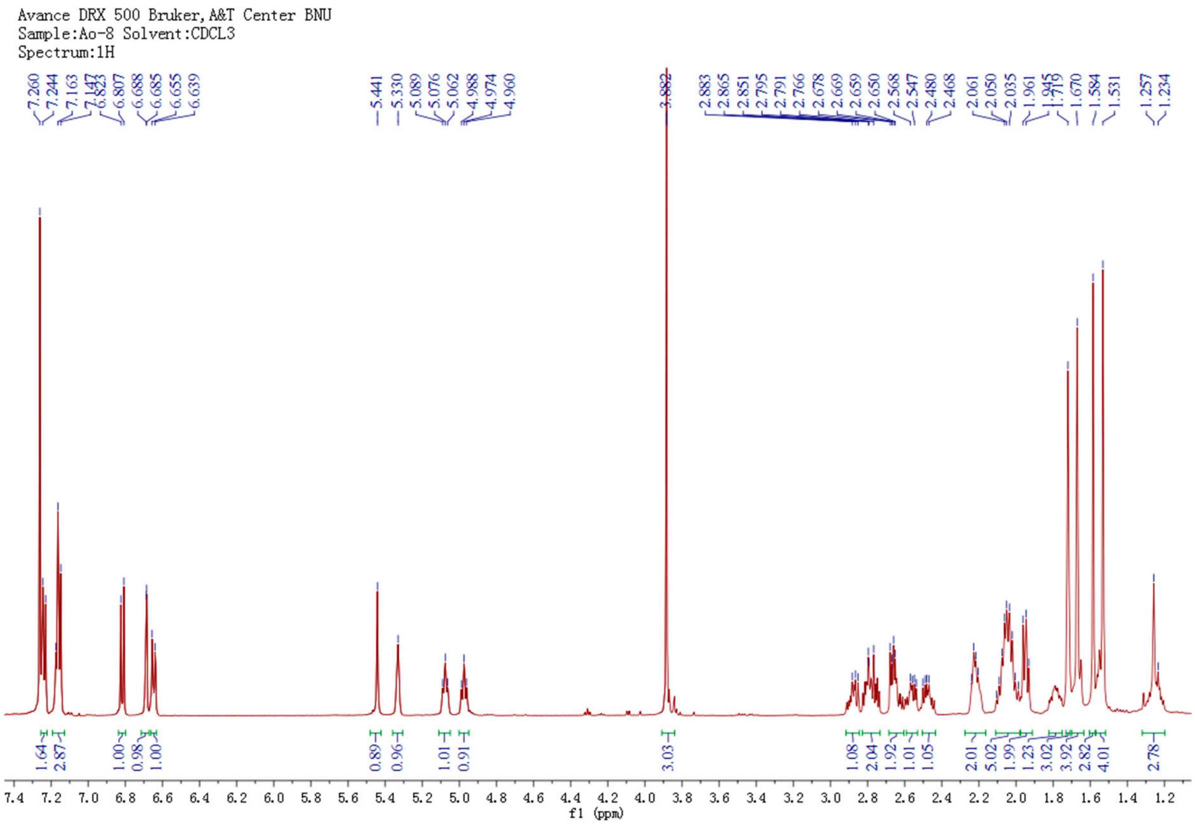

Figure 1S. ^{1}H-NMR spectrum of compound **3** ($CDCl_3$).

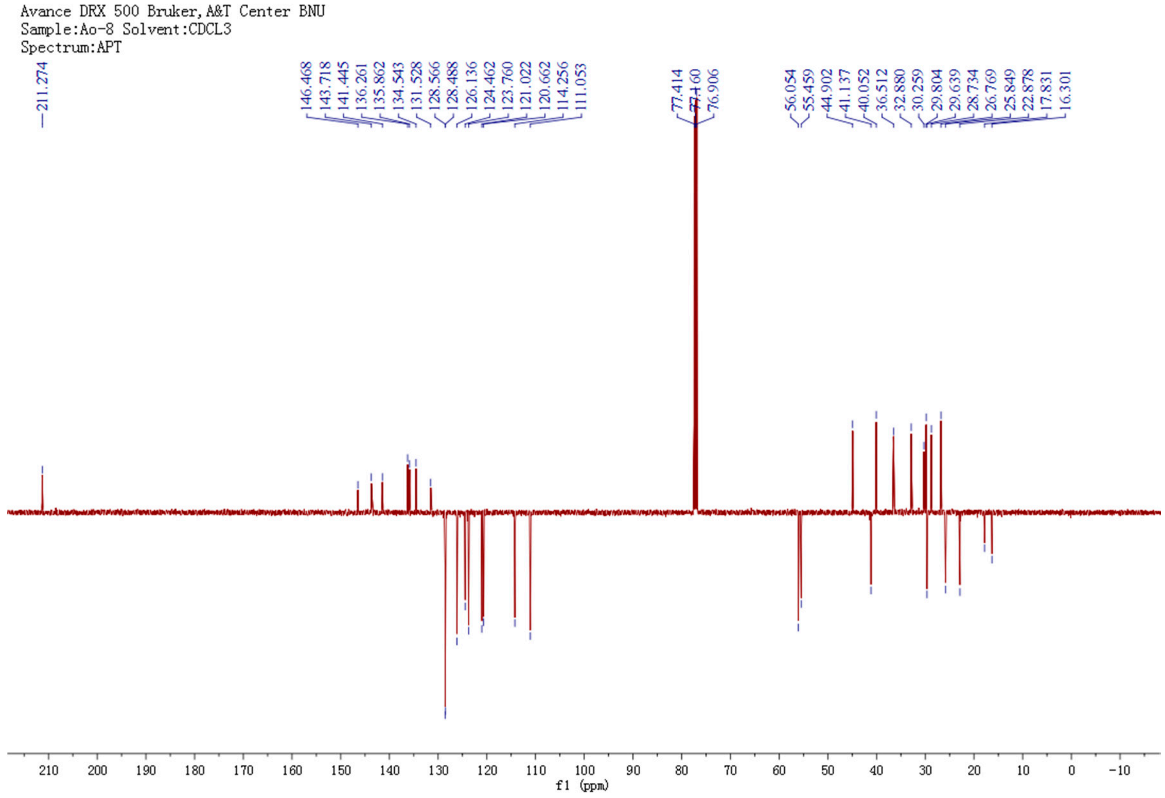

Figure 2S. APT spectrum of compound **3** ($CDCl_3$).

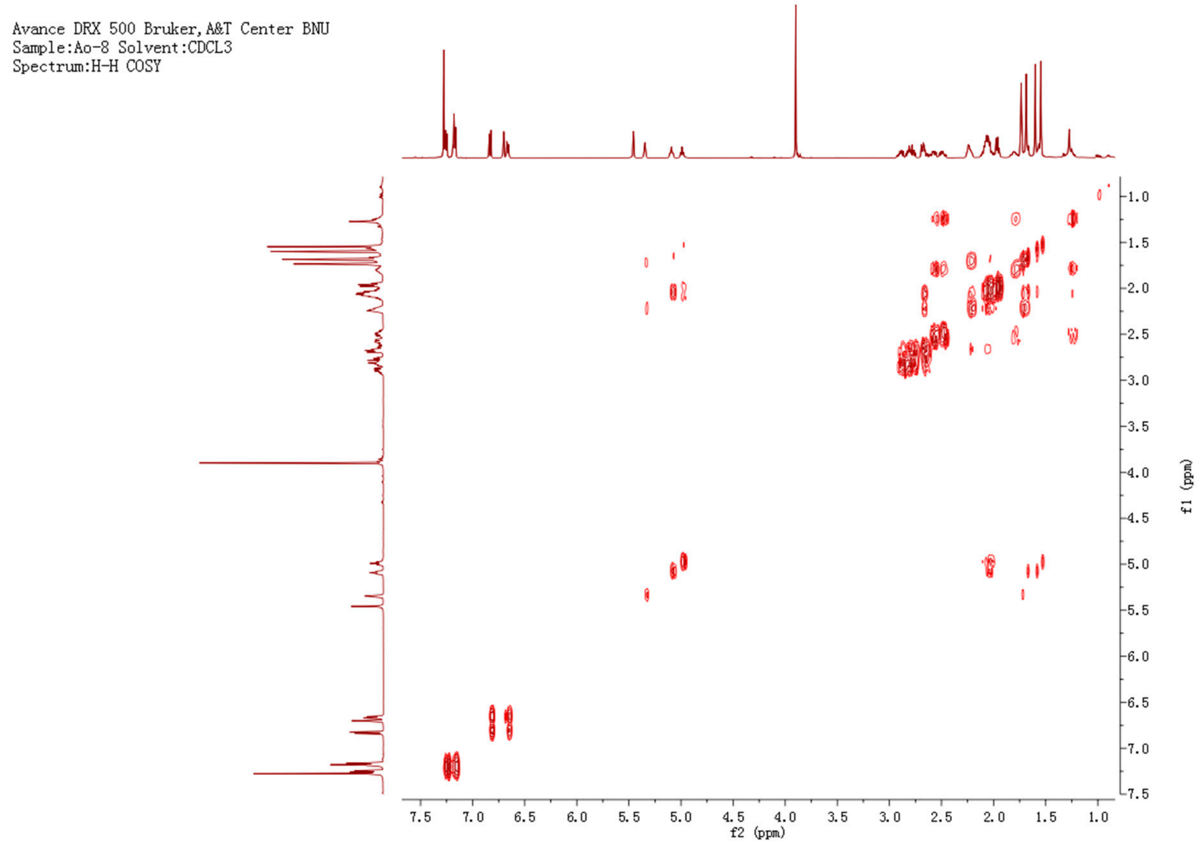

Figure 3S. H-H COSY spectrum of compound **3** ($CDCl_3$).

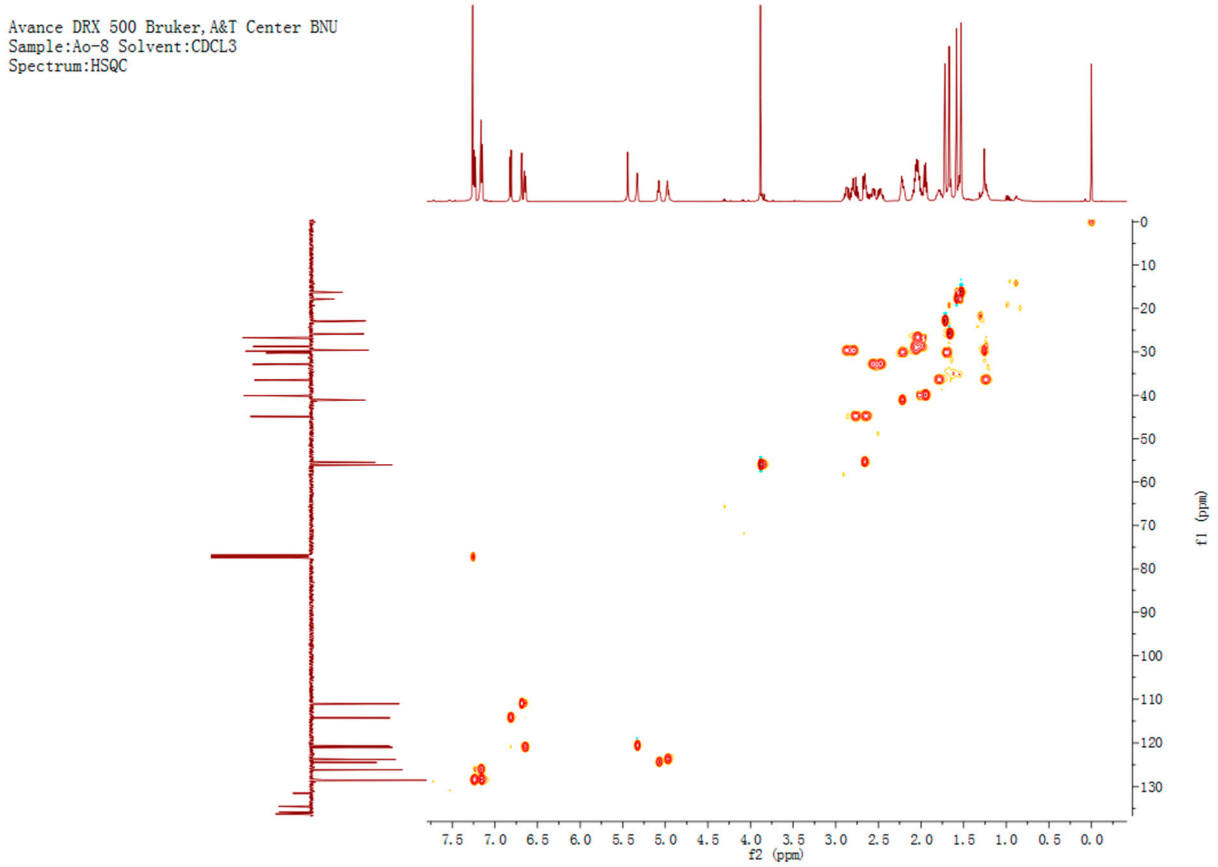

Figure 4S. HSQC spectrum of compound **3** ($CDCl_3$).

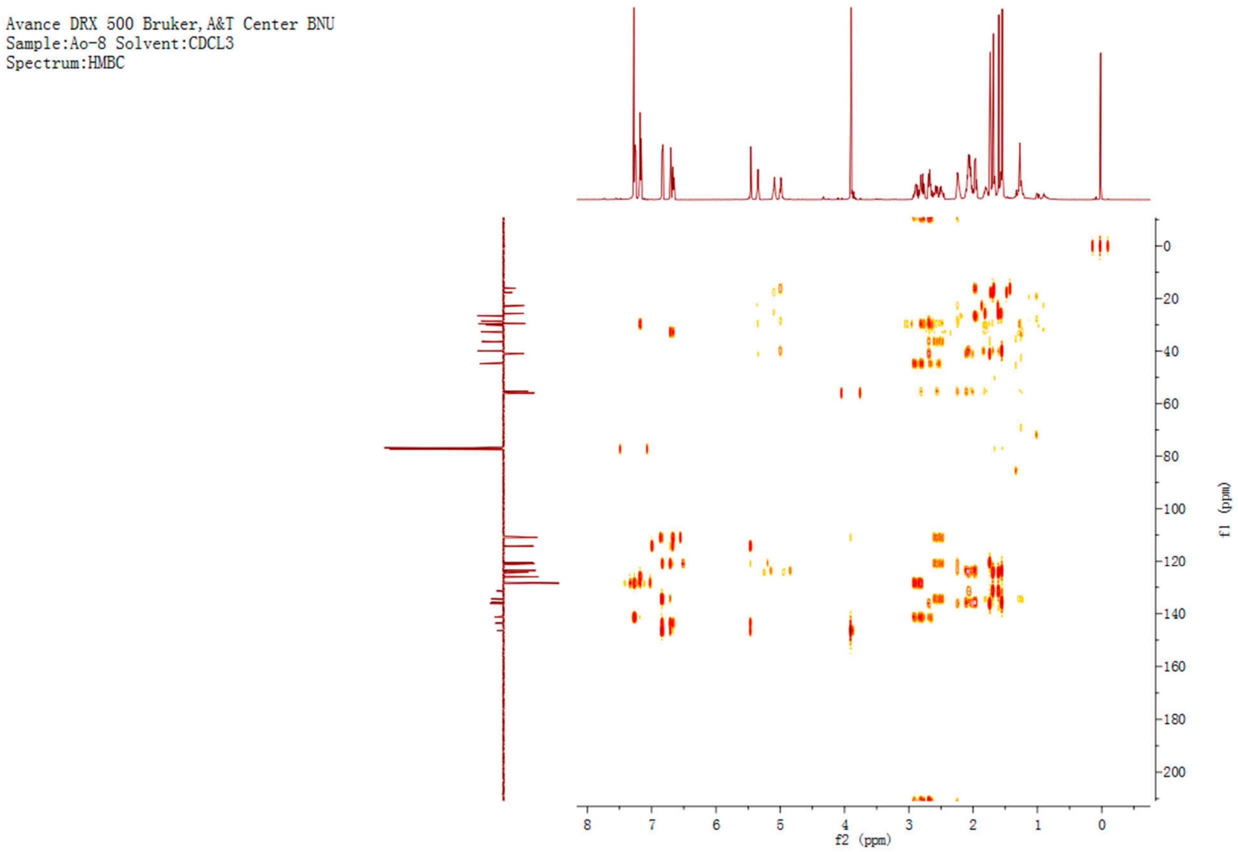

Figure 5S. HMBC spectrum of compound **3** ($CDCl_3$).

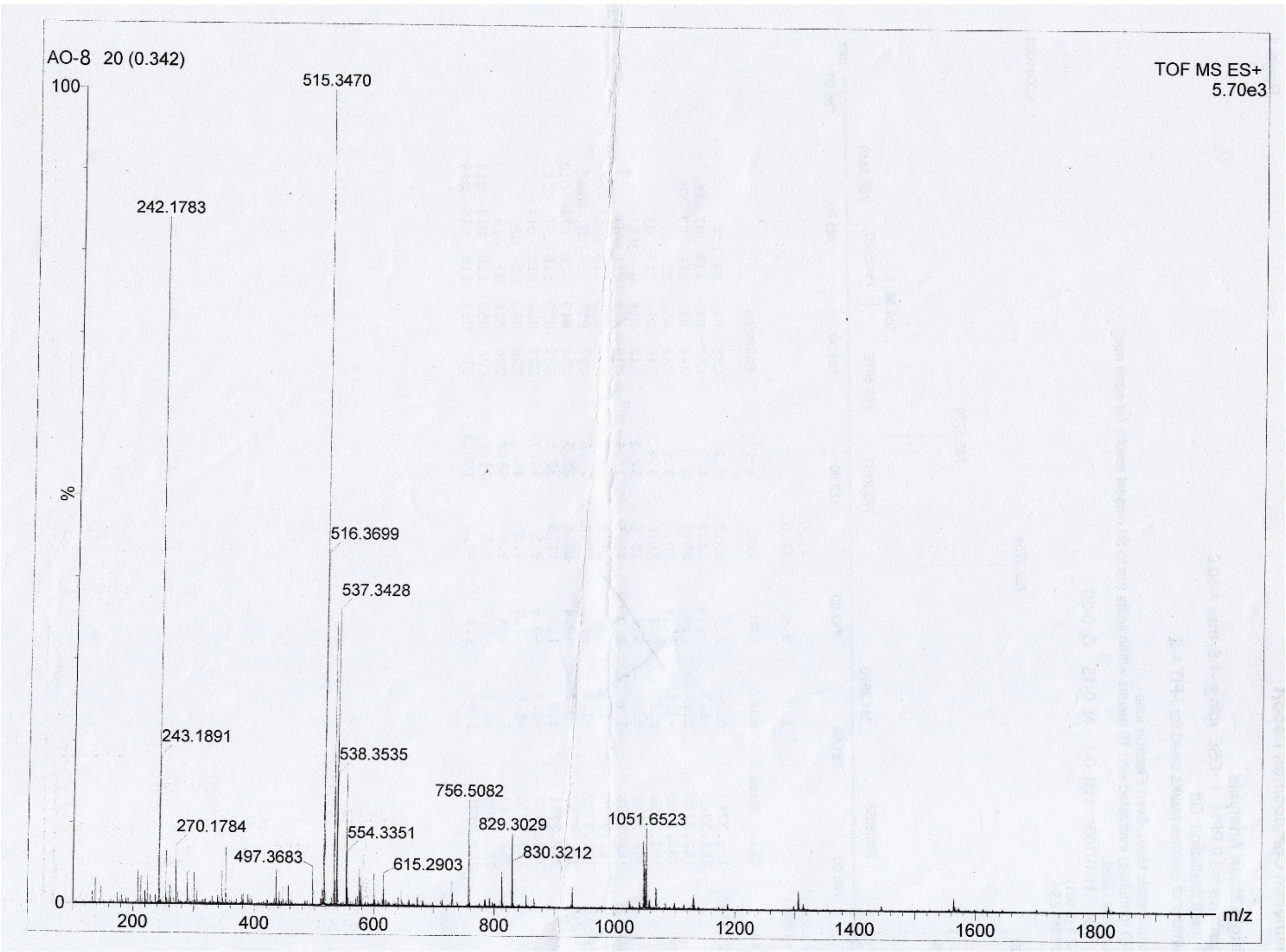

Figure 6S. ESIMS spectrum of compound **3** ($CDCl_3$).
